# miR-29b attenuates tumorigenicity and stemness maintenance in human glioblastoma multiforme by directly targeting *BCL2L2*

**DOI:** 10.18632/oncotarget.4384

**Published:** 2015-06-19

**Authors:** Hyun Joo Chung, Young Eun Choi, Eun Sook Kim, Young-Hoon Han, Myung-Jin Park, In Hwa Bae

**Affiliations:** ^1^ Division of Radiation Cancer Research, Korea Institute of Radiological & Medical Sciences, Seoul, Korea; ^2^ Research Center for Radio-Senescence, Korea Institute of Radiological & Medical Sciences, Seoul, Korea

**Keywords:** miR-29b, BCL2L2, tumorigenicity, stemness, GBM

## Abstract

Glioblastoma multiforme (GBM) is the most common malignant brain tumor and exhibits aggressive and invasive behavior. We previously identified four miRNAs—miR-29b, 494, 193a-3p, and 30e—with enhanced expression in GBM following treatment of ionizing radiation by miRNA microarray analysis. In this study, we found that only miR-29b inhibited tumor cell migration and invasion by reducing MMP-2 activity via phospho-AKT/β-catenin signaling, and stimulated a more epithelial-like morphology. Moreover, miR-29b inhibits angiogenesis by attenuating tube formation and the expression of VEGF and Ang-2, and stemness maintenance in GBM cells, as demonstrated by decreasing neurosphere formation and cancer stem cell marker protein expression. These findings support the anti-tumor properties of miR-29b in human GBM cells. Furthermore, miR-29b expression was inversely proportional to that of *BCL2L2* mRNA or protein in various cancer cell types. Interestingly, *BCL2L2* mRNA is highly expressed in the mesenchymal type of GBM. To further elucidate the relationship between miR-29b and *BCL2L2* in GBM, we performed co-transfection reporter assays and determined that miR-29b downregulates *BCL2L2* expression by directly binding its 3′UTR. Finally, we confirmed that *BCL2L2* repression is of central importance to miR-29b anti-tumor activity using functional assays to examine cell migration, invasion, angiogenesis, and stemness. From these data, we propose that miR-29b may be a useful therapeutic agent in GBM.

## INTRODUCTION

Glioblastoma multiforme (GBM) is the most common and aggressive form of astrocyte-derived brain malignancy [1]. Patients diagnosed with GBM are often given a poor prognosis due to considerable difficulties in radical resection, owing to its highly invasive growth and high rate of recurrence [2, 3].

Current evidence suggests that the B-cell lymphoma 2 homolog, BCL2-like 2 (*BCL2L2*; also known as Bcl-w) enhances tumorigenicity and cell survival through its anti-apoptotic activity in cancer cells [4–7]. *BCL2L2* is expressed in various cancer types-including gastric cancer, colorectal adenocarcinomas, and GBM-in a cancer cell-specific manner. We previously reported that *BCL2L2* enhances the migratory and invasive potentials of gastric cancer cells by facilitating the production of several types of extracellular matrix (ECM)-degrading proteinases [8, 9]. In addition, *BCL2L2* potentiates aggressiveness, stemness, and a mesenchymal phenotype [10] in glioblastoma cells by inducing the nuclear translocation of β-catenin [11]. These observations support the claim that *BCL2L2* overexpression is strongly correlated with tumorigenicity in GBM.

MicroRNAs (miRNAs) comprise, a class of small RNA molecules approximately 18–24 nucleotides in length, which negatively regulate gene expression by directly binding to complementary sequences in the 3′-untranslated region (3′UTR) of target mRNAs. miRNAs function as guides for post-transcriptional gene silencing, producing sequence-specific mRNA cleavage, or translational repression that can elicit a notable effect on cellular phenotype [12, 13]. Some miRNAs act as tumor suppressors [14–16] or oncogenes, depending on the function of their target genes [17, 18]. In particular, miR-29b expression enhances the survival of patients with hepatocellular carcinoma (HCC) by repressing matrix metalloproteinase 2 (MMP-2) expression and activity. Thus, miR-29b might present as a promising HCC therapy [19]. Furthermore, forced miR-29b expression has been shown to abrogate myeloid cell leukemia-1 (MCL1) protein expression in human cholangiocarcinoma cells [20].

In previous study, we examined changes in the miRNA expression profile of U251 GBM cells in response to treatment with ionizing radiation (IR) by miRNA microarray analysis [21]. Subsequent functional analyses revealed that only miR-29b attenuates cell migration and MMP-2 activity; thus, we used an miR-29b mimic to investigate functions of miR-29b as tumor suppressor in GBM cells. Notably, miR-29 family members (miR-29a, -29b, and -29c) indirectly activate the p53 tumor suppressor by targeting p85α (the regulatory subunit of PI3K), resulting in cancer cell apoptosis [22]. Additionally, miR-29 has been reported as tumor suppressor to target oncogenes such as T-cell leukemia/lymphoma 1 (TCL1) [23, 24] and myeloid leukemia cell differentiation protein 1 (MCL1) [20].

In this study, we demonstrate that *BCL2L2* is an oncogenic, miR-29b target gene and is upregulated in the mesenchymal subtype of GBM. To our knowledge, no study has examined the relationship between miR-29-regulated *BCL2L2* and the malignant phenotype of GBM cells. Therefore, we propose that miR-29b has potential as an anti-cancer therapy in GBM by targeting oncogenic *BCL2L2*.

## RESULTS

### miRNAs which screened by ionizing radiation evaluate migratory potential and MMP-2 activity in U251 cells

**Figure 1:**
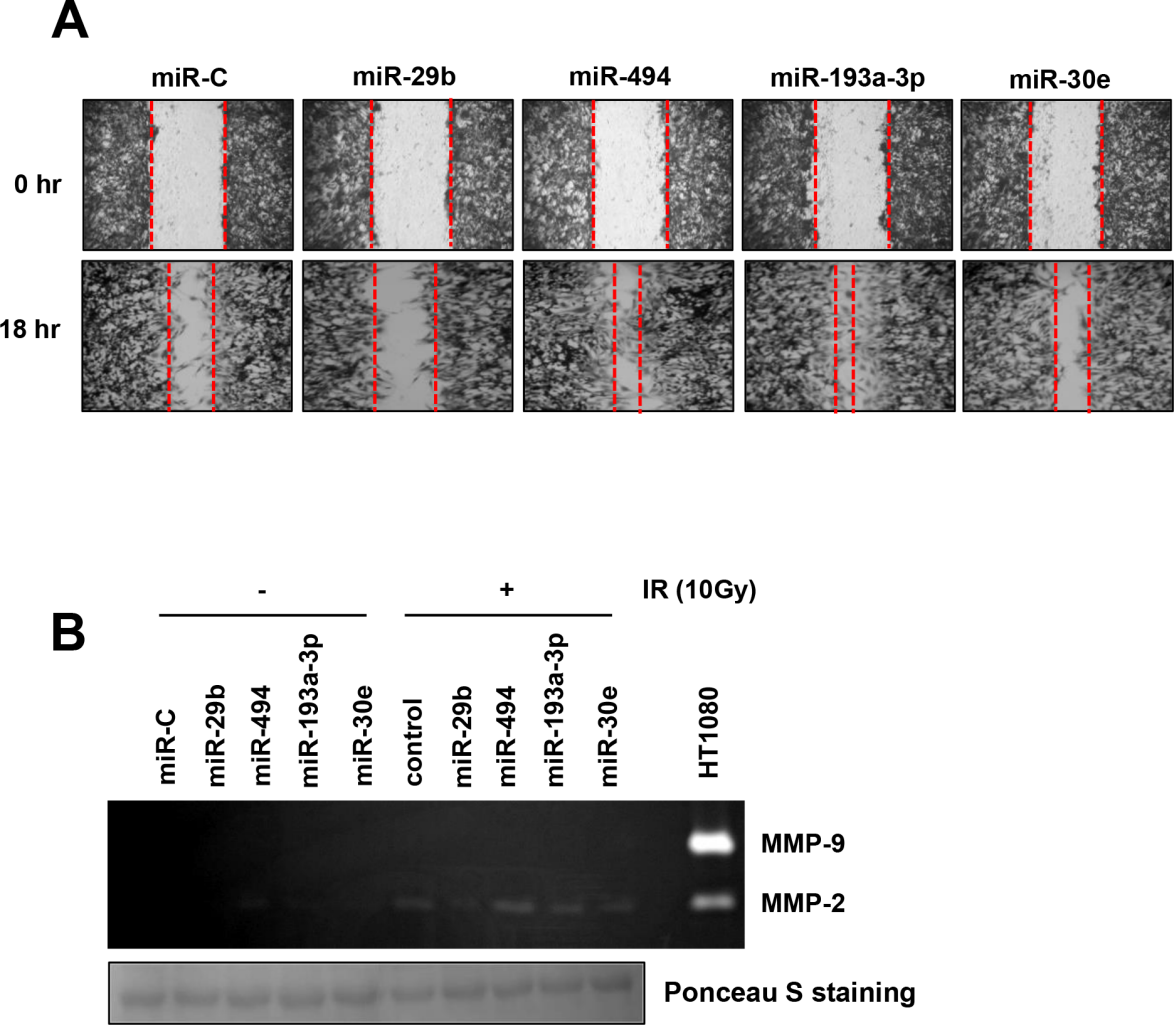
IR-related miRNAs regulate migratory potential and MMP-2 activity in U251 cells **A**. U251 cells were transfected with 20 nM of miR-29b, miR-494, miR-193a-3p or miR-30e and cell migration was evaluated by wound healing assay 18 hr later. **B**. U251 cells were transfected with four synthetic miRNAs and treated with or without 10 Gy ionizing radiation. Supernatants were harvested 24 hr later in serum free media to examine MMP-2/9 activity by gelatin zymography. HT1080 fibrosarcoma cell supernatant was used as a positive control. Ponceau S staining serves as a loading control.

We identified miRNAs in U251 GBM cells that were significantly up- or downregulated after treatment with IR by miRNA microarray analysis in previous study [21]. From the data generated, we selected miR-29b, 494, 193a-3p, and 30e, which exhibited a >1.5-fold change in expression in response to IR treatment compared to that observed in control cells [21]. To select miRNAs downregulated in the aggressive phenotype of human GBM, we individually evaluated their role in cell migration and MMP-2/9 activity, using wound healing assays and gelatin zymography, respectively. As a result, only miR-29b could sufficiently inhibit both migratory potential (Figure 1A) and MMP-2 activity (Figure 1B). Meanwhile, we confirmed that MMP-9 activity was not induced by miRNA mimics or IR (10 Gy) in U251 cells using HT1080 fibrosarcoma cells as a positive control. In turn, we have selected miR-29b to investigate functions of miR-29b as tumor suppressor in GBM cells.

### miR-29b limits the migratory and invasive capacities in U251 and U87MG GBM cells by inhibiting MMP-2 activity and protein expression

**Figure 2:**
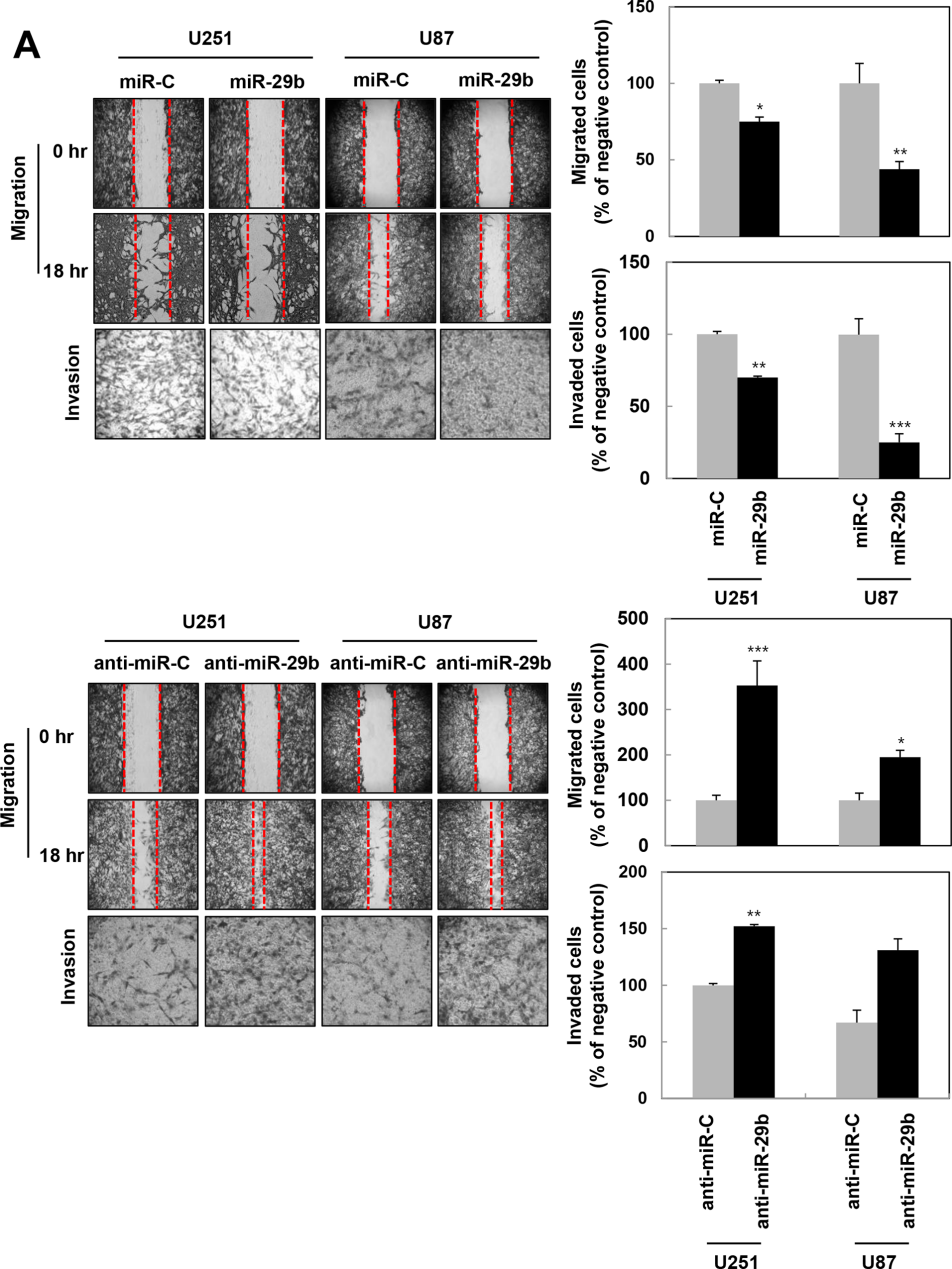

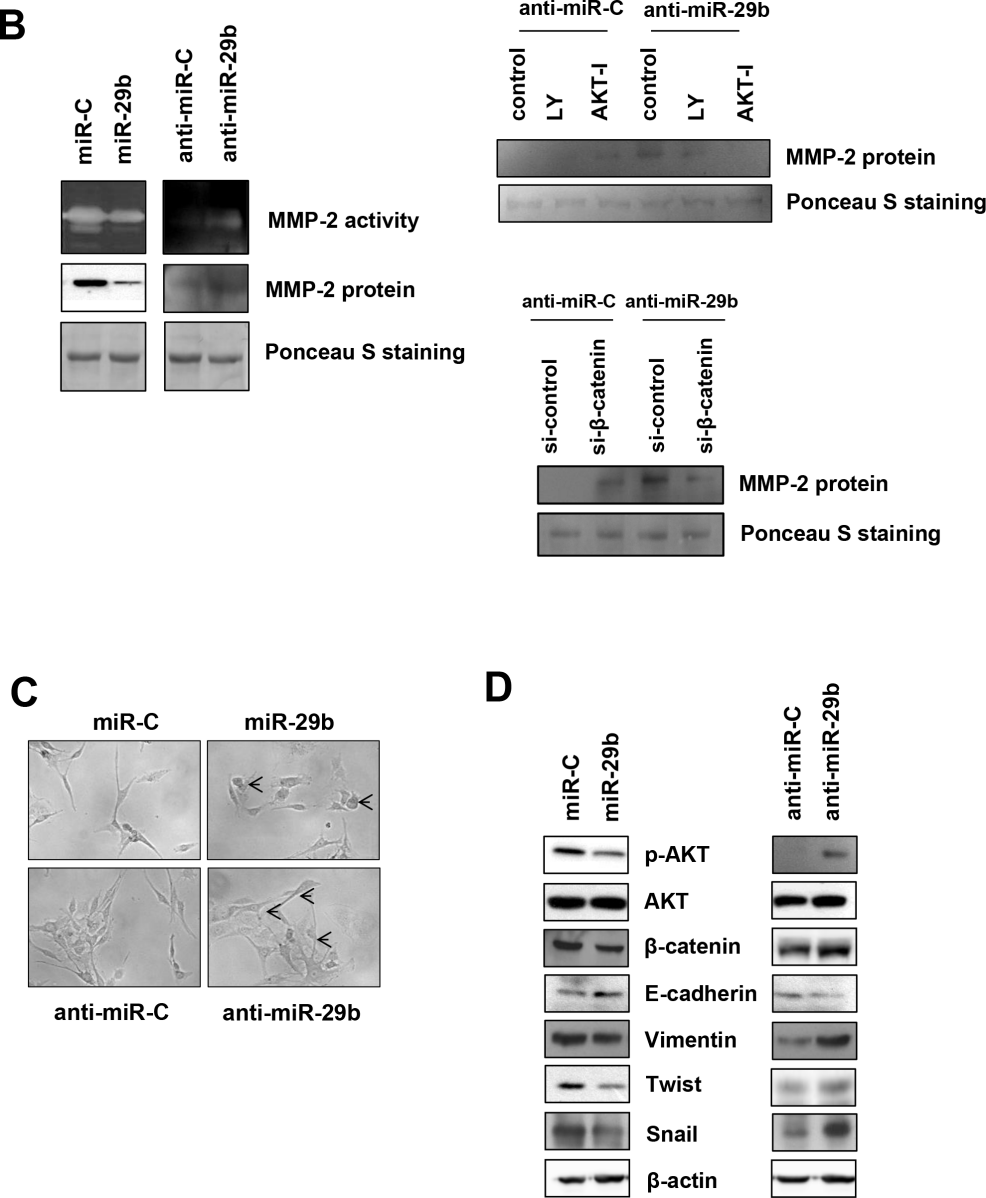
miR-29b limits cell migration and invasion by abrogating MMP-2 activity and mesenchymal traits via AKT-β-catenin signaling, respectively **A.** miR-29b inhibits the migratory potential and invasiveness of U251 and U87MG glioblastoma cells. Cells were quantified in three fields from the scratched area (200 × 500 μm^2^) under a light microscope after transfection with scrambled control microRNA (miR-C) and synthetic miR-29b (Top) or anti-miR-C and miR-29b inhibitor (bottom). The transfected cells were seeded onto Matrigel-coated polycarbonate membranes and invasive cells were stained and counted under a light microscope. Data represents the mean ± SD of three independent experiments; *, *p* < 0.05; **, *p* < 0.005. **B**. Left, MMP-2 enzymatic activity and protein level were measured by gelatin zymography and western blotting, respectively. Bottom, Ponceau S used as a load-control staining. Right, MMP-2 protein levels were also examined after transfection of anti-miR-C and anti-miR-29b (20 nM) in response to treatment with PI3K (LY294002) or AKT (AKT-I) inhibitors for 1 hr, or co-transfection with siRNA β-catenin (10 nM). Bottom, Ponceau S load-control staining. **C.** U251 morphology was observed under a light microscope 48 hr after transfection (200 ×). Arrowheads denote rounded miR-29b mimic-transfected cells and adherent fibroblastic-like anti-miR-29b-transfected cells. **D.** Levels of phosphorylated AKT (p-AKT), AKT, PTEN, β-catenin, and epithelial- or mesenchymal-specific markers, such as E-cadherin, Vimentin, Twist, and Snail were detected by western blotting. The function of miR-29b mimic was confirmed using an anti-miR-29b. β-actin was used as a loading control.

To characterize the function of miR-29b in cancer cells, U251 cells were transiently transfected with synthetic miR-29b or miR-29b inhibitor and miR-29b expression was confirmed by quantitative real-time PCR (data not shown). As expected, cells transfected with synthetic miR-29b exhibited a significant drop in their migratory and invasive capacities when compared to scrambled control miRNA (miR-C)-transfected cells by wound healing and Matrigel invasion assays, respectively, as confirmed in both U251 and U87MG GBM cells (Figure 2A). In contrast, cells treated with miR-29b inhibitor displayed slightly enhanced migration and invasion when compared to that observed in negative control (anti-miR-C)-treated U251 and U87MG cells (Figure 2A). Moreover, we assessed MMP-2 activity and protein expression in U251 cells by gelatin zymography and western blotting, respectively, after transfection with synthetic miR-29b or inhibitor and found that both were significantly suppressed by the miR-29b mimic, but enhanced by transfection of miR-29b inhibitor (Figure 2B). To confirm that these events regulated MMP-2, we assessed MMP-2 protein expression levels by immunoblotting following treatment with PI3K inhibitor (LY294002), AKT inhibitor (AKT-I), or β-catenin siRNA. As a result, miR-29b inhibitor-induced MMP-2 protein levels were downregulated in response to all three, thus confirming that the synthetic miR-29b mimic downregulated MMP-2 protein levels by attenuating AKT/β-catenin signaling (Figure 2B right). In wound healing assays (Figure 2A), we found that the wound margins in miR-29b-overexpressing cells displayed a rounded shape when compared to miR-29b inhibitor-transfected counterparts, which changed the fibroblast-like morphology as indicated by the arrowheads (Figure 2C). Accordingly, we investigated EMT-related protein expression and found that miR-29b attenuated mesenchymal marker expression, including Vimentin, Twist, and Snail, while enhancing that of the epithelial marker, E-cadherin. Conversely, the miR-29b inhibitor increased the expression of mesenchymal-related proteins (Figure 2D). Therefore, these data revealed that miR-29b potentiates mesenchymal-to-epithelial transition in GBM cells.

To better describe the tumor suppressive signaling of miR-29b mimic in PTEN-mutant U251 cells, we examined the expression levels of proteins known to regulate this transition-including phosphorylated AKT (p-AKT), AKT, and ß-catenin in cells transfected with miR-29b mimic or miR-29b inhibitor by western blotting (Figure 2D). Notably, miR-29b prevented both AKT activation and β-catenin expression (Figure 2D). In contrast, miR-29b inhibitor-transfected cells elevated mesenchymal marker expressions and diminished E-cadherin expression, as well as enhanced AKT activation and β-catenin expression. Altogether, these data confirmed that miR-29b attenuated the mesenchymal properties of GBM cells by decreasing MMP-2 activity through Akt/ß-catenin signaling.

### miR-29b attenuates tumor angiogenesis

**Figure 3:**
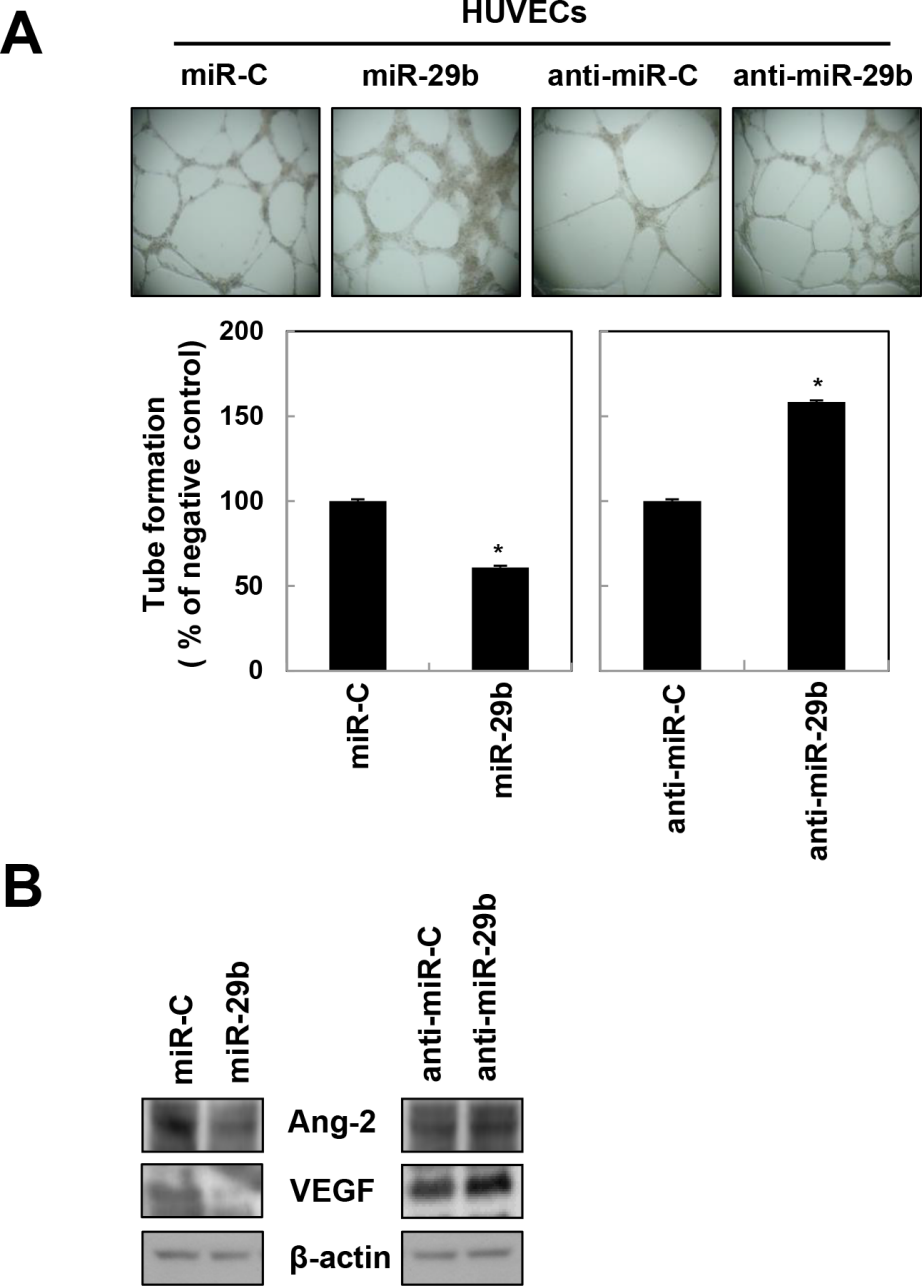
miR-29b attenuates angiogenesis in HUVECs **A.** miR-29b downregulates tube formation in HUVECs. HUVECs were transfected with miR-C, miR-29b, anti-miR-C, or anti-miR-29b, seeded onto Matrigel-coated 96 well plates for 24 hr later (1 × 10^4^ cells/well), and then monitored for 8 hr to assess tube formation. **B.** U251 cells were transfected with indicated miRNAs were measured the expression of angiogenesis-related proteins such as angiopoietin 2 (Ang-2) or vascular endothelial growth factor (VEGF), using western blotting. β-actin was used as a loading control. *, *p* < 0.05.

To determine if miR-29b was also sufficient to regulate angiogenesis, human umbilical vein endothelial cells (HUVECs) and human brain microvascular endothelial cells (HBMECs) were transfected with miR-29b mimic or inhibitor. Notably, the miR-29b mimic decreased angiogenesis by attenuating tube formation in both HUVECs and HBMECs (Figure 3A and data not shown); as well as expression of the angiogenesis-related factors, angiopoietin-2 (Ang-2) and vascular endothelial growth factor (VEGF) in U251 cells (Figure 3B). In contrast, miR-29b inhibitor-transfected HUVECs displayed increased tube formation (Figure 3A), as well as Ang-2 and VEGF expression (Figure 3B). These findings support that miR-29b can also suppress GBM aggressiveness by attenuating angiogenesis.

### miR-29b inhibits the maintenance of stemness

**Figure 4:**
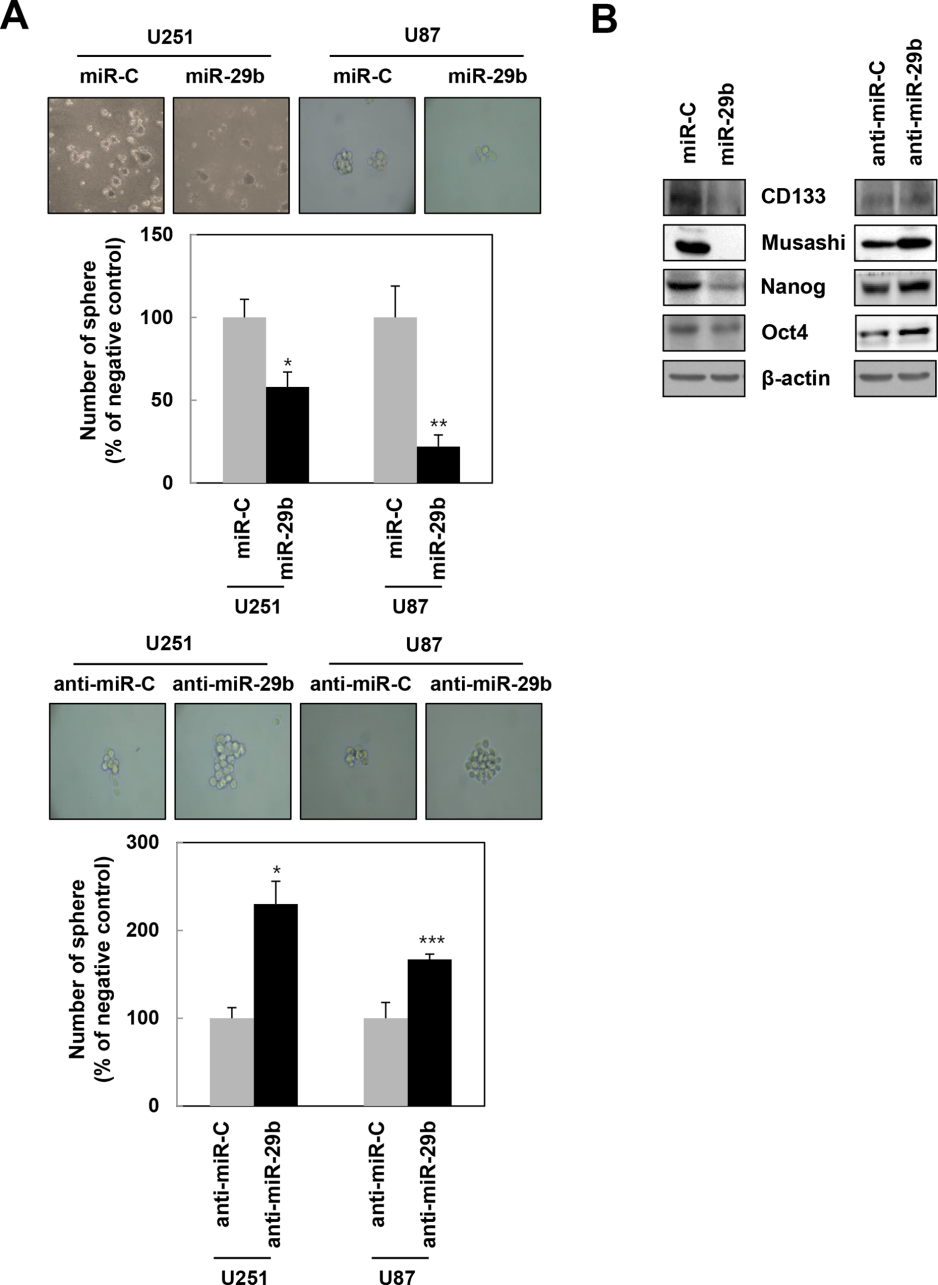
miR-29b regulates the maintenance of stemness in GBM cells **A.** U251 and U87MG cells were transfected with synthetic miR-29b (top) or anti-miR-29b (bottom) seeded onto 100 mm culture dishes (1 × 10^3^ cells/dish), and cultured for 7~10 days to generate neurospheres. *, *p* < 0.05. **B.** The expression of cancer stem cell marker proteins, including CD133, Musashi, Nanog, and Oct4, were then monitored in U251 cells by western blotting, with β-actin as a loading control.

Neurosphere formation is one method to assess the self-renewal capacity of neuronal stem cells and their derivatives. Indeed, both U251 and U87MG miR-29b-overexpressing cells demonstrated a dramatic decrease in neurosphere formation when compared to anti-miR-controls. In contrast, cells transfected with miR-29b inhibitor showed an increased potential for neurosphere formation (Figure 4A). Moreover, the miR-29b mimic downregulated the expression of cancer stem-like marker proteins, including CD133, Musashi, Nanog, and Oct4, in U251 cells. Accordingly, transfection with the miR-29b inhibitor rescued the expressions of these proteins dramatically (Figure 4B). Altogether, these results suggest that miR-29b likely functions to promote stemness maintenance in GBM cells.

### *BCL2L2* is a direct target of miR-29b

**Figure 5:**
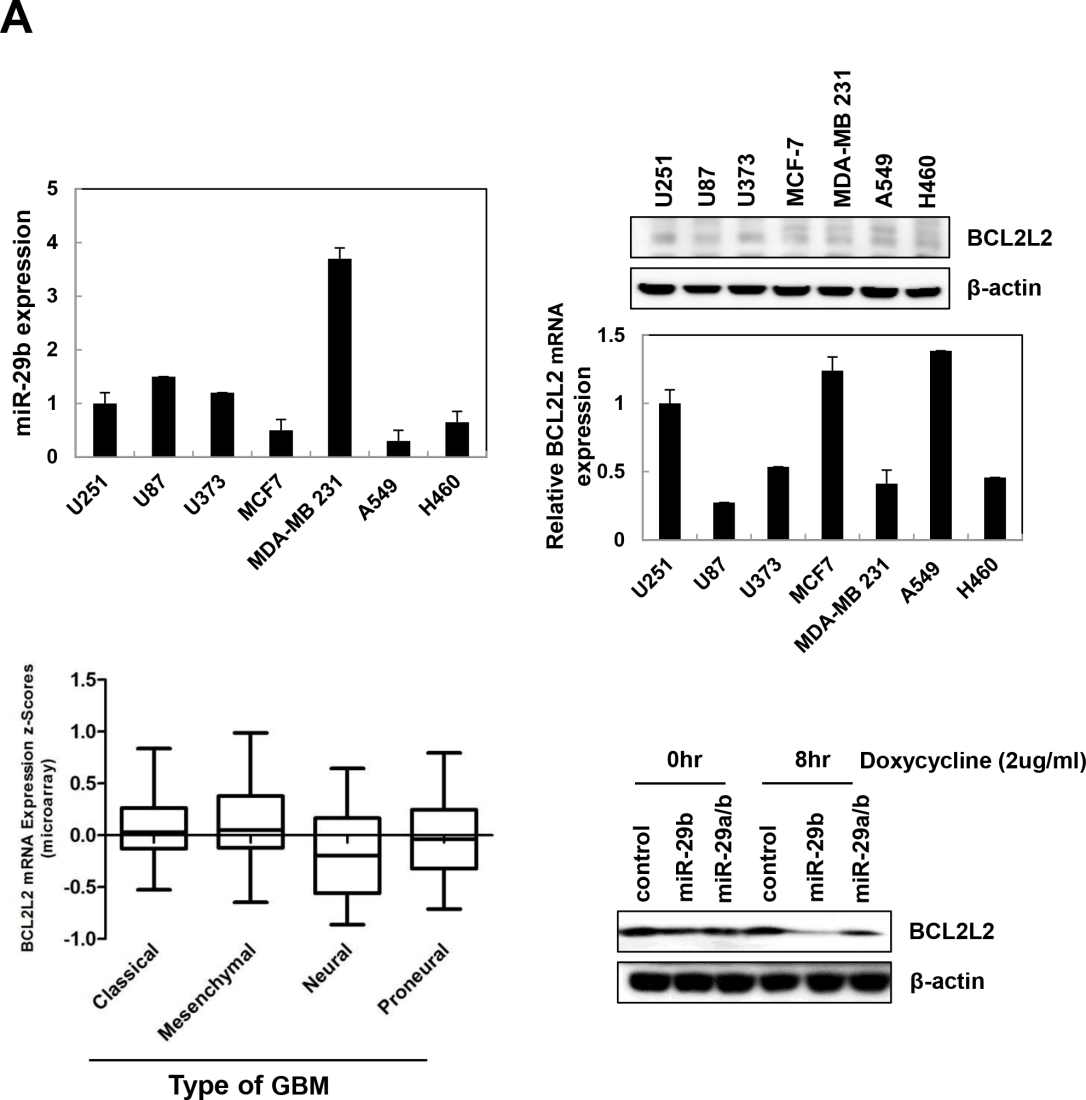

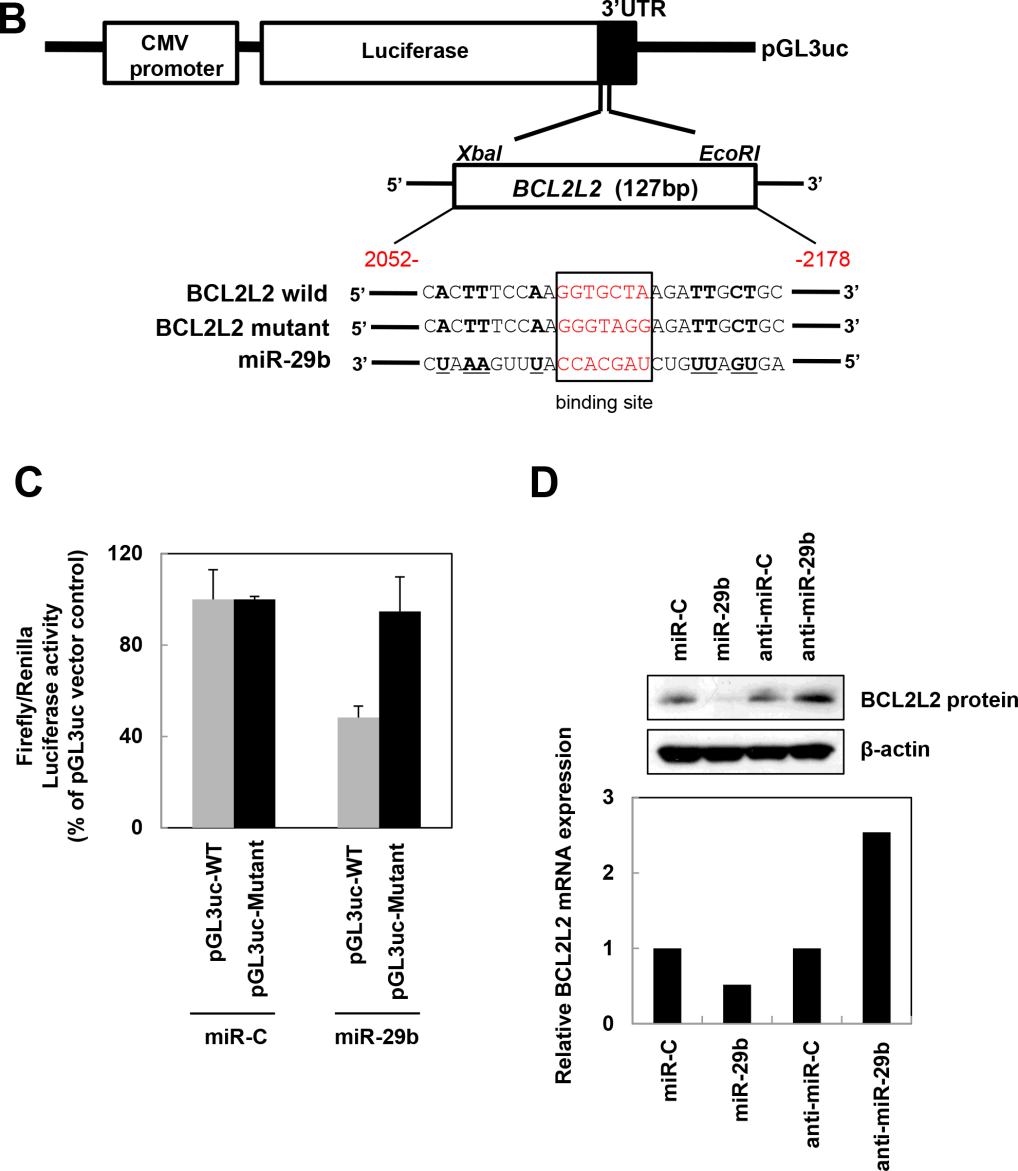
*BCL2L2* is a direct target gene of miR-29b **A.** miR-29b (top left) and *BCL2L2* protein or mRNA expression levels (top right) were monitored by quantitative real-time PCR or western blotting, respectively, in the following cancer cell lines: U251, U87MG, U373 (glioma); MCF7, MDA-MB-231 (breast); A549 and H460 (lung). Bottom left, *BCL2L2* mRNA expression in GBM subtypes based on clinical information available in the TCGA (The Cancer Genome Atlas). Bottom right, *BCL2L2* protein expression in U251 cells with Tet-inducible expression of the miR-29 cluster (miR-29a/b) or miR-29b alone. **B.** Structure of reporter constructs containing *BCL2L2* 3′-untranslated region (3′UTR) downstream of the luciferase ORF. **C.** Luciferase activity was monitored in transfected cells. Luciferase activity was normalized to that of firefly luciferase using the *Renilla*-TK vector. Data are the combined results from three independent experiments. **, *p* < 0.005. **D.** *BCL2L2* protein or mRNA levels were measured by qRT-PCR or western blot analysis in U251 cells transfected with synthetic miR-29b or anti-miR-29b. Anti-miR-C was used as an inhibitor of miR-C.

To identify miR-29b target genes that act as tumor suppressors, we questioned oncogenic genes highly expressed in tumor patients using miRNA site prediction (www.microrna.org; www.targetscan.org; www.mirdb.org) and TCGA (The Cancer Genome Atlas) databases. This search revealed the *BCL2L2* oncogene as an miR-29b target gene upregulated in the mesenchymal subtype of GBM (Figure 5A, bottom left). Therefore, we focused our investigations on *BCL2L2* in order to determine miR-29b function. To first confirm the relationship between miR-29b and *BCL2L2*, we measured miR-29b and *BCL2L2* protein or mRNA levels by quantitative real-time PCR and immunoblotting in various cancer cells, including gliomas (U251, U87MG, and U373) breast (MCF7 and MDA-MB-231), and lung (A549 and H460) cancer cell lines, and found that miR-29b was relatively downregulated in all except MDA-MB 231 cells (Figure 5A, top left). Meanwhile, *BCL2L2* protein or mRNA was highly expressed in U251, MCF-7, and A549 cells (Figure 5A, top right). These analyses revealed an inverse correlation between miR-29b expression and *BCL2L2* mRNA or protein level, supporting the notion that *BCL2L2* is a target of miR-29b. To further expand on the clinical significance of this finding, clinical data evaluating *BCL2L2* mRNA expression was accessed from The Cancer Genome Atlas (TCGA) GBM dataset. Significantly, when GBM tumors were classified into classical, mesenchymal, proneural, and neural types, *BCL2L2* mRNA expression was the highest in the mesenchymal subtype (Figure 5A, bottom left). This supported our previous studies defining *BCL2L2* as an oncogene that promotes the aggressiveness of GBM. To determine if the miR-29b alone was more effective than the full miR-29a/b cluster, we evaluated *BCL2L2* protein expression in a Tet-on inducible system with miR-29b or miR-29a/b cluster (Figure 5A, bottom right) [22]. Interestingly, while both miR-29b and miR-29a/b suppressed *BCL2L2* protein expression, miR-29b alone was more effective in inhibiting *BCL2L2*. Accordingly, we only used miR-29b mimic in the subsequent studies (Figure 5A, bottom right).

We next used luciferase assays to further elucidate the relationship between miR-29b and *BCL2L2*. First, we constructed pGL3UC-*BCL2L2* vectors containing the wild-type miR-29b binding site (5′-GGTGCTA-3′, *BCL2L2*-WT) or a non-binding mutant (5′-GGGTAGG-3′, *BCL2L2*-mutant) (Figure 5B), and then co-transfected U251 cells with miR-C or miR-29b and either pGL3uc-*BCL2L2*-WT, *BCL2L2*-mutant, or the empty vector for 48 hr. Notably, miR-29b/pGL3uc-*BCL2L2*-WT co-transfectants exhibited lower luciferase activity, whereas no differences were observed between the pGL3uc-*BCL2L2*-mutant and empty control vector (Figure 5C). These data demonstrate that miR-29b directly binds the 3′UTR of *BCL2L2* and inhibits protein and mRNA expression through sequence-specific mRNA cleavage (Figure 5D).

### miR-29b-dependent *BCL2L2* gene regulation attenuates GBM tumorigenicity

**Figure 6:**
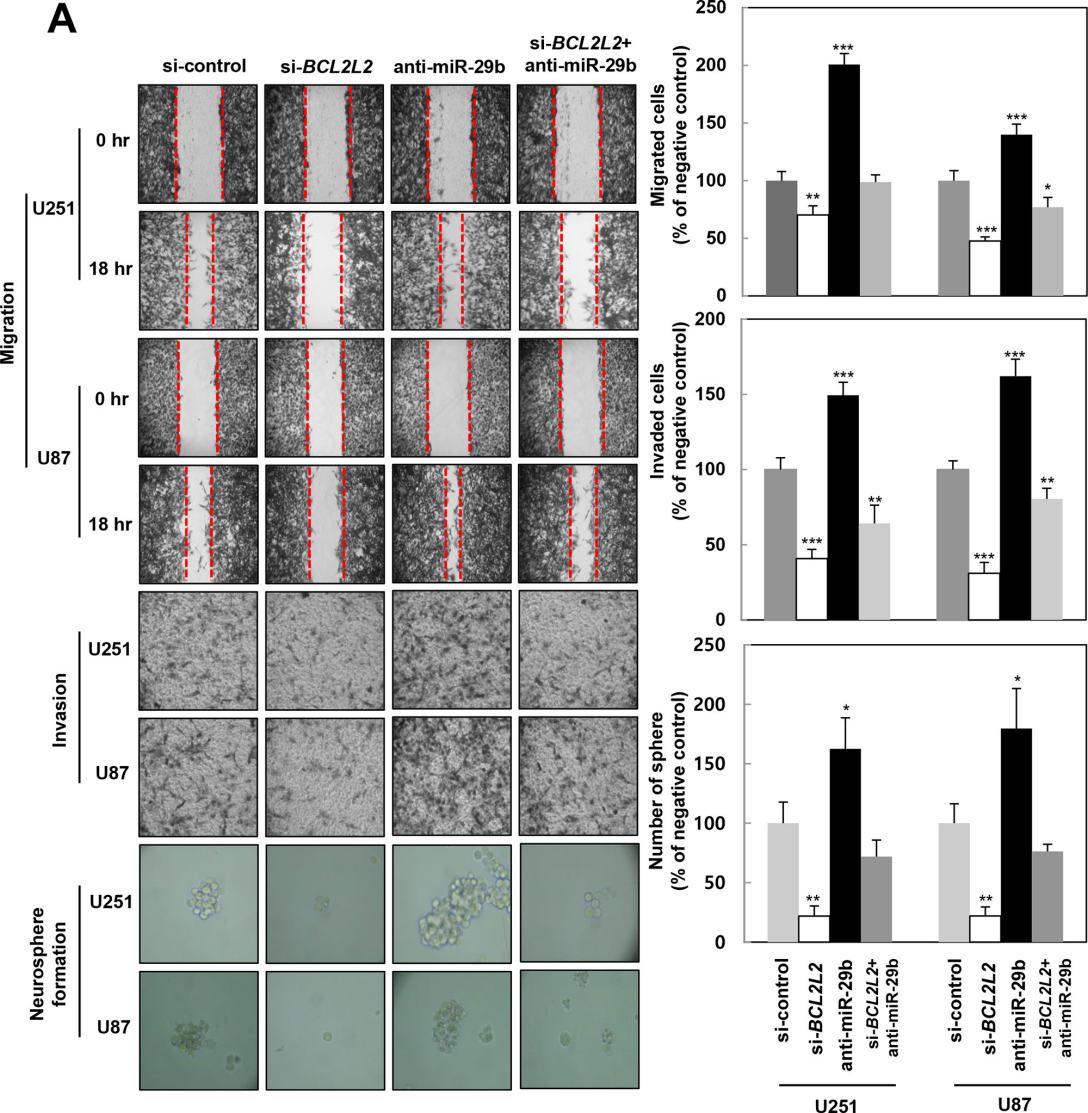

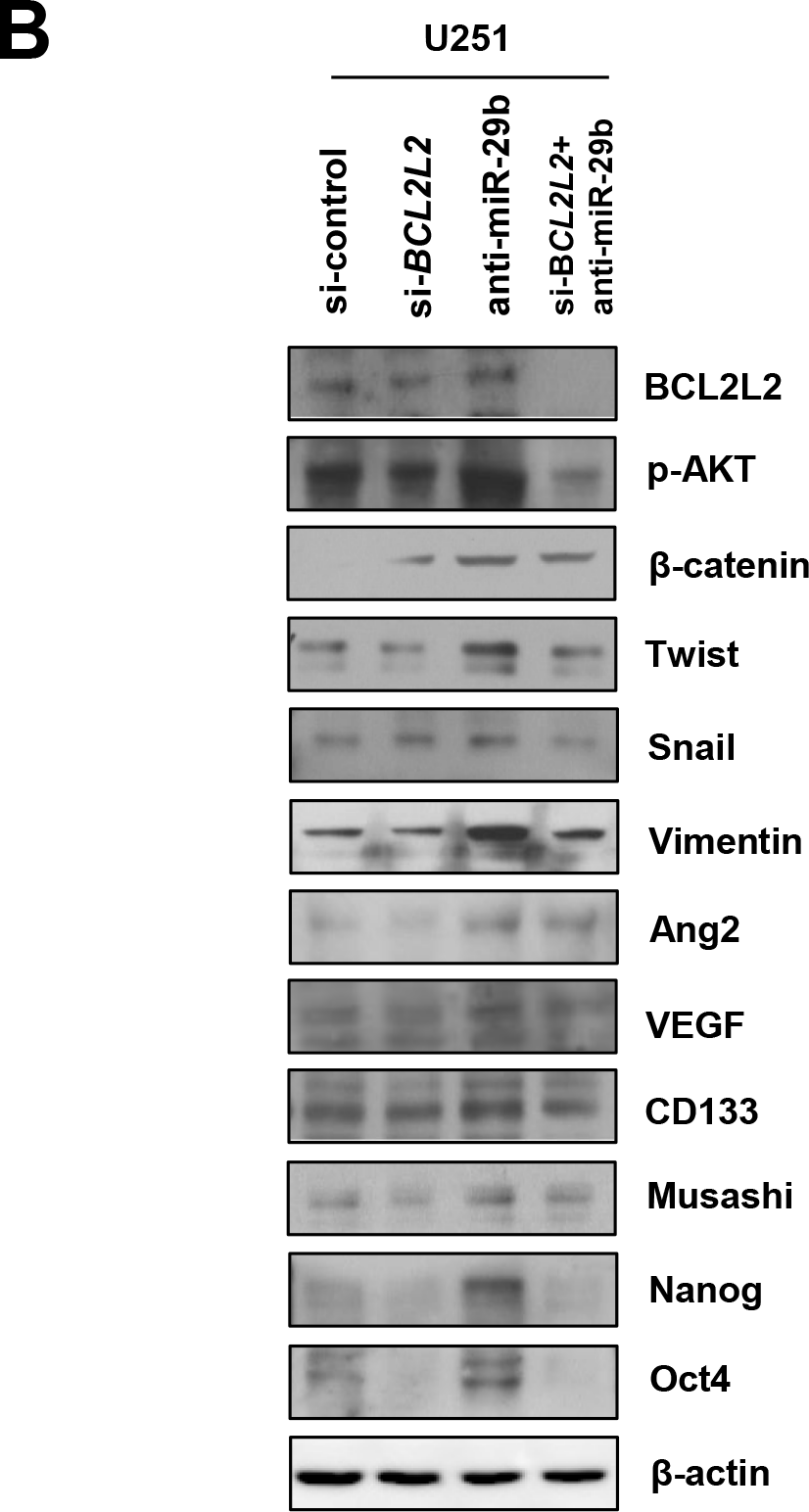
miR-29b directly decreases malignant actions of *BCL2L2* in GBM cells **A.** Transfected cells with si-control, si-*BCL2L2*, synthetic anti-miR-29b and si-*BCL2L2*+anti-miR-29b were conducted by migration for 18 hr, Matrigel invasion assays for 18 hr, and neurosphere formation assay for 5 days in U251 and U87MG cells. *, *p* < 0.05, **, *p* < 0.005. **B.** Levels of p-AKT, AKT, β-catenin, mesenchymal-related proteins, cancer stem cell markers, angiogenic factors, and *BCL2L2* were measured by western blot analysis, with β-actin for load controls.

**Figure 7:**
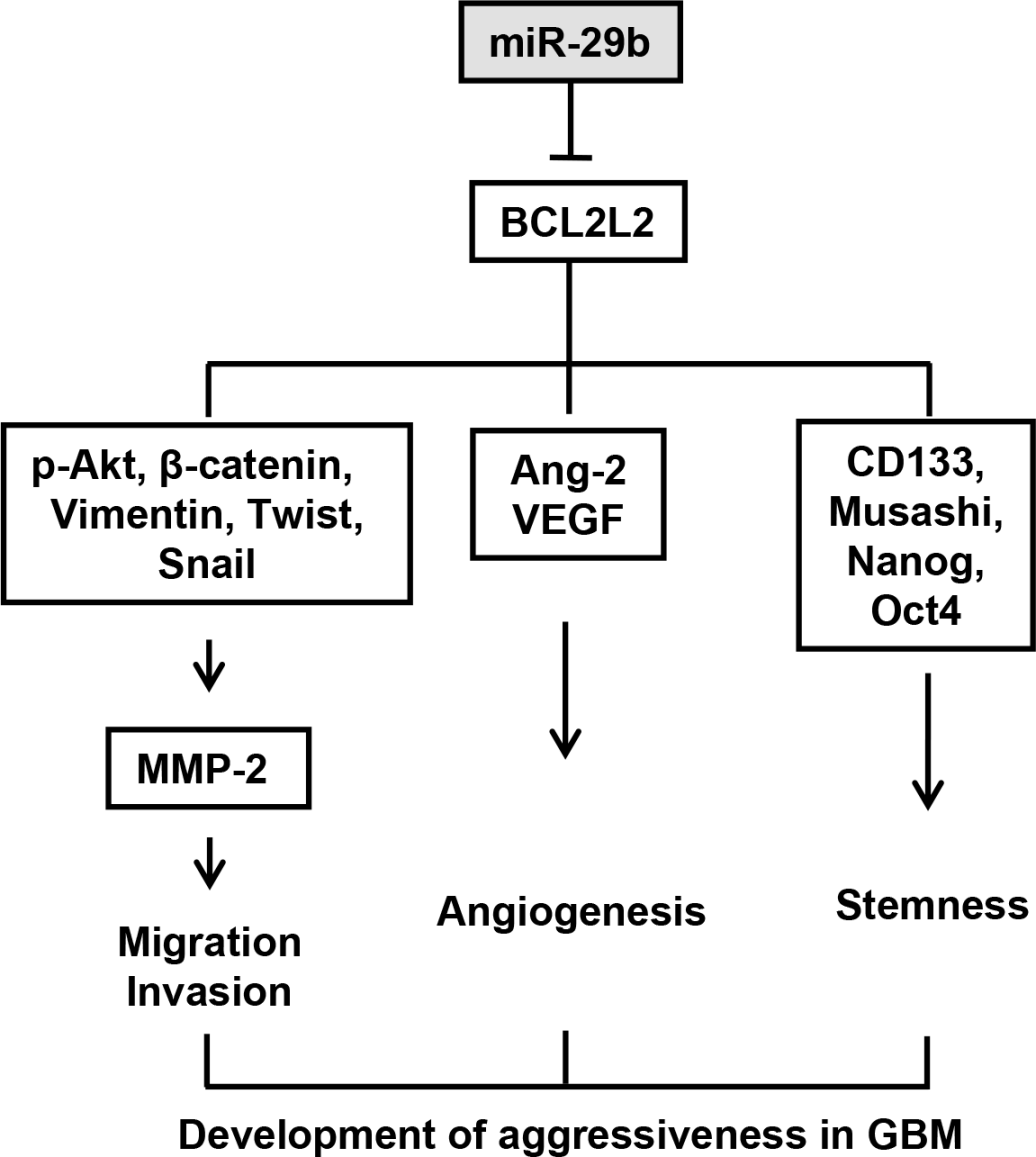
Mechanistic summary for tumorigenicity and stemness maintenance inhibiting actions of miR-29b through the direct targeting of *BCL2L2* mRNA in GBM miR-29b attenuates migration, invasion, and mesenchymal morphology by inactivating MMP-2 and decreasing Ang-2 and VEGF expression. Concurrently, miR-29b inhibits the maintenance of stemness as demonstrated by defects in neurosphere formation and cancer stem cell marker proteins expression. These phenomena demonstrate that miR-29b directly targets and represses oncogenic *BCL2L2* expression. Therefore, miR-29b may be useful as therapeutic agent by attenuating aggressiveness of GBM.

To verify the above data, we examined whether miR-29b regulated the migratory potential, invasiveness, angiogenesis, and stemness maintenance of GBM by repressing *BCL2L2*. Thus, U251 and U87MG cells transfected with si-control, si-*BCL2L2*, anti-miR-29b, or co-transfected with si-*BCL2L2* and anti-miR-29b were subjected to migration, invasion, and neurosphere formation assays (Figure 6A), and immunoblotting (Figure 6B). Notably, si-*BCL2L2*-transfected cells exhibited a decreased capacity for migration, invasion, angiogenesis, and stemness, similar to that observed in miR-29b-overexpressing cells. In contrast, miR-29b inhibitor-transfected cells displayed increased migration and invasion via activating p-AKT-ß-catenin signaling. Moreover, these cells were increased the formation of neurospheres, indicative of increased stemness that was later confirmed by cancer stem cell marker protein expression analysis (Figure 6A, 6B). Meanwhile, si-*BCL2L2*/anti-miR-29b-co-transfected cells were blocked oncogenic functions of anti-miR-29b by decreasing anti-miR-29b-induced migration, invasion, angiogenesis, and stemness, and exhibited no significant differences in comparison to si-*BCL2L2-*transfected cells. These data confirmed that miR-29b represses *BCL2L2* expression by directly binding its 3′UTR of *BCL2L2* mRNA, and described their functional relationship in greater detail. Altogether, these analyses support the notion of a molecular system whereby miR-29b inhibits aggressive GBM development via *BCL2L2* inhibition, and implicate miR-29b as a potential candidate in anti-cancer therapy (Figure 7).

## DISCUSSION

An extensive amount of research has led to the identification of miRNA as important regulators of gene expression in cancer biology [12, 18]. The expression of miRNAs can vary by cancer types and plays a critical role in cell survival, apoptosis [20], proliferation, cell death, and tumorigenesis [18, 25]. Meanwhile, ionizing radiation (IR) is often used as a therapeutic modality in addition to chemotherapy and surgery [26–28]. Our group previously analyzed IR-induced miRNA expression changes by miRNA microarray analysis [21]. From this data, we identified miR-29b, 494, 193a-3p, and 30e as IR-regulated miRNAs that exhibited a > 1.5 fold change in treated U251 GBM cells compared to untreated controls [21]. Matrix metalloproteinases (MMPs), including MMP-2 and MMP-9, facilitate the migratory or invasive potential of tumor cells by enzymatically degrading the extracellular matrix [8, 19]. To investigate miRNAs downregulated in the aggressive phenotype of human GBM, we evaluated the effect of these four miRNAs on cell migration and MMP-2/9 activity in U251 glioblastoma cells and found that only miR-29b markedly decreased these characteristics (Figure 1A, 1B). Thus, we decided to investigate the functions of miR-29b as tumor suppressor in GBM. According to previous reports, miR-29b suppresses angiogenesis, invasion, and metastasis by inhibiting MMP-2 expression in hepatocellular carcinoma [19]; however, to our knowledge, no study has examined the relationship between miR-29 and *BCL2L2* expression and their effect on GBM cell phenotype. To characterize the function of miR-29b, U251 and U87MG GBM cells were transiently transfected with synthetic miR-29b or miR-29b inhibitor, which revealed that miR-29b markedly hindered their migratory and invasive capacities when compared to a scrambled miRNA-transfected cells (miR-C) (Figure 2A, top) by inhibiting MMP-2 activity and protein expression (Figure 2B). In contrast, miR29b inhibitor-transfected cells displayed a slight enhancement in these characteristics when compared to that observed in counterparts of negative control (anti-miR-C) by augmenting MMP-2 activity (Figure 2A, bottom). To identify the intracellular signaling pathway that mediate this effect, we examined MMP-2 protein levels following treatment with PI3K inhibitor (LY294002), AKT inhibitor (AKT-I), or siRNA β-catenin, and showed that all three significantly attenuated MMP-2 protein levels. Thus, we confirmed that synthetic miR-29b mimic reduced the migratory and invasive capacity of cells by attenuating MMP-2 protein expression via AKT-β-catenin signaling (Figure 2B, right).

Epithelial-mesenchymal transition is characterized by the loss of cell-cell adhesion, cytoskeletal rearrangement, and the manipulation of mesenchymal properties, and is a crucial step in cancer metastasis [29, 30]. Thus, EMT facilitates important cellular actions associated with tumorigenicity and metastasis in various tumors. In our wound healing assays, we found that the wound margin in miR-29b-overexpressing cells displayed a rounded shape compared to that of miR-29b inhibitor-transfected counterparts, which exhibited a more fibroblast-like morphology (Figure 2C). In response to this finding, we assessed the expression of EMT-related proteins and found that miR-29b attenuated the expression of the mesenchymal markers Vimentin, Twist, and Snail, while enhancing that of the epithelial marker, E-cadherin. Contrarily, miR-29b inhibitor increased the expression of these mesenchymal-related proteins. Therefore, these data indicated that miR-29b potentiates mesenchymal-to-epithelial transition by decreasing MMP-2 activity via AKT/ß-catenin signaling in U251 cells (Figure 2D).

It was previously reported that miR-29b-expressing hepatocellular carcinoma (HCC) cells exhibit significantly lower microvessel densities and intrahepatic metastasis in animal models [19]. Accordingly, we found that treatment with synthetic miR-29b effectively attenuated angiogenesis by inhibiting tube formation in HUVECs (Figure 3A) and HBMECs (data not shown), as well as expression of the angiogenic factors Ang-2 and VEGF (Figure 3B). These findings are consistent with earlier studies reporting that miR-29b suppresses the invasive and angiogenic potentials of HCC cells [19]. Moreover, in acute myeloid leukemia (AML), restoration of miR-29b in AML cell lines and primary samples resulted in the onset of apoptosis and dramatically reduced cellular tumorigenicity in a xenograft leukemia model [23, 24], which were supported other reports citing that synthetic miR-29b regulates apoptosis, cell cycle progression, and proliferation in leukemia cells [31]. In addition, miR-29b suppresses metastasis by targeting GATA3, which promotes epithelial-mesenchymal transition in breast cancer [32]. Concurrently, miR-29b potentiates cancer cell apoptosis by targeting p85α and CDC42 to subsequently activate the p53 tumor suppressor [22]. We also showed that miR-29b inhibits tumorigenicity in GBM cells as defined by their capacities for migration, invasion, and self-renewal. The ability for cells to maintain a cancer stem cell-like property, or “stemness, “ was assessed by neurosphere formation assays and cancer stem-like cell (CSC) marker expression analysis. Significantly, miR-29b-transfected U251 and U87MG cells displayed defects in their ability to form neurospheres (Figure 4A), as well as a reduced expression of the CSC marker proteins CD133, Musashi, Nanog, and Oct4 (Figure 4B).

Anti-tumor miRNAs inhibit the expression of genes involved in angiogenesis, extracellular matrix signaling, and metastasis [32–35]. To identify miR-29b target genes that act as tumor suppressors, we searched for oncogenic factors that are highly expressed in tumor patients using miRNA identification algorithms and TCGA (The Cancer Genome Atlas) GBM dataset. To confirm this prediction, we had measured miR-29b and *BCL2L2* protein or mRNA levels in various cancer cells and noted an inverse relationship between the two (Figure 5A), which supported *BCL2L2* as a direct target of miR-29b. To further expand the clinical significance of this finding, we analyzed the differences in *BCL2L2* mRNA expression in various GBM subtypes and found a significant upregulation specifically in mesenchymal type tumors. This supported our hypothesis for *BCL2L2* in promoting cancer cell migration, invasion, and EMT [10, 11, 36].

To further elucidate the relationship between miR-29b and *BCL2L2*, we performed luciferase assays *BCL2L2*-expressing vectors with a wild-type or mutated miR-29b binding site (Figure 5B), and found the miR-29b expression had no effect on luciferase activity in cells transfected with the mutant site (Figure 5C). These data demonstrate that miR-29b directly binds the *BCL2L2* 3′UTR to inhibit its expression (Figure 5D).

Moreover, we confirmed that si-*BCL2L2*-transfected cells displayed attenuated migration, invasion, angiogenesis, and stemness similar to that observed in miR-29b overexpressing cells, while miR-29b inhibitor counterparts displayed enhanced migration and invasion associated with the increased phosphorylation of AKT and expression of β-catenin (Figure 6). Meanwhile, no changes were observed in si-*BCL2L2*/anti-miR-29b-co-transfected cells compared to si-*BCL2L2*-transfected cells, presumably because si-*BCL2L2* blocked others effects of anti-miR-29b (Figure 6).

Altogether, these data reveal a crucial role for miR-29b in limiting the aggressive phenotype of glioblastoma (Figure 7). Based on our findings, we propose that miR-29b might be useful as an anti-cancer therapeutic agent in GBM.

## MATERIALS AND METHODS

### Cell culture and reagents

U251, U87, and U373 (glioma); MCF7 and MDA-MB-231 (breast); A549 and H460 (lung) bought from Korea Cell Line Bank (KCLB). U251 and U87 cells cultured in Minimum Essential Medium Eagle (MEM, Mediatech, Inc., Manassas, VA), U373 and MCF7 and MDA-MB-231 cells cultured in DMEM media (Mediatech, Inc., Manassas, VA) and A549 and H460 cultured in RPMI 1640 media (Mediatech, Inc., Manassas, VA). HUVECs cultured in endothelial cell growth medium MV2 with supplementmix (Promo Cell GmbH, Heidelberg, Germany). All medium supplemented with 10% heat-inactivated fetal bovine serum and 0.1% penicillin-streptomycin antibiotics (PAA Laboratories GmbH, Pasching, Austria) in a humidified 5% CO_2_ incubator at 37°C. Antibodies against *BCL2L2*, p-AKT, AKT, β-catenin, and Musashi were purchased from Cell Signaling technology (Beverly, MA). Ang-2 and VEGF antibodies were purchased from Santa Cruz Biotechnology (Santa Cruz, CA), and Snail and Oct-4 antibodies were from Novus Biologicals (Littleton, CO). The MMP-2 antibody was purchased from Calbiochem (La Jolla, CA), E-cadherin antibody was from BD Transduction Laboratories (San Jose, CA), Vimentin antibody was from Thermo Fisher Scientific (Fremont, CA), Twist antibody was from Abcam Inc. (Cambridge, MA), Nanog antibody was from Chemicon International (Temecula, CA), CD133 antibody was from MiltenyiBiotec Inc. (Auburn, CA), and β-actin antibody was from Sigma-Aldrich (St Louis, MO). PI3K inhibitor (LY294002) and Akt inhibitor were purchased from Calbiochem (La Jolla, CA). siRNA *BCL2L2* and β-catenin were from Santa Cruz Biotechnology (Santa Cruz, CA).

### Plasmid DNAs

To prepare the reporter construct, a DNA fragment of human *BCL2L2* 3′UTR containing the putative miR-29b binding site (127 bp) was amplified by PCR, and cloned into pGL3uc. The nucleotide sequences of primers for the amplification of the *BCL2L2* 3′UTR were 5′-TGAAGCCACACTGTTTGCAT-3′ (forward) and 5′-GAGAA TTCTTAGCATTGGAGAG-3′ (reverse), which contained the binding site of miR-29b (GGTGCTA). The vector of mutant type is constructed out of “GGGTAGG” that have site directed mutagenesis of the *BCL2L2* 3′UTR for binding of miR-29b. Then PCR product and pGL3uc vector were cut by *Xba*I and *EcoR*I restriction enzymes and fragment of PCR product was ligated into pGL3uc vector. pGL3uc-*BCL2L2* plasmid transformed into *Ecoli* and cultured at 37°C for overnight by shaking.

To do functional analyses, cell were transiently transfected with the vector control, *BCL2L2* and a Tet-on inducible system with pTRE2 hyg-miR-29b or pTRE2 hyg-miR-29b-1~29a cluster expression constructs for 48 hrs using Lipofectamine 2000 (Invitrogene, Carlsbad, CA) according to the manufacturer's recommendation. pGL3uc vector and pTRE2 hyg-miR-29b or pTRE2 hyg-miR-29b-1~29a cluster expression constructs kindly provide by V. N. Kim (School of Biological Sciences, Seoul National University, Korea) [22].

### RNA oligoribonucleotides and transfection

Synthetic miRNA mimics were synthesized by Samchully Pharmaceutical (Seoul, Korea) as RNA duplexes designed from the sequences of miR-29b (5′-UAGCAC CAUUUGAAAUCAGUGUU-3′), miR-494 (5′-UGAAACAUACACGGGAAACCUC-3′, miR-193a-3p (5′-AACUGGCCUACAAAGUCCCAGU-3′), and miR-30e (5′-UGUAAACAUCCUUGACUGGAAG-3′ using 5′-UGAAUUAGAUGGCGAUGU UTT-3′ for the control. The inhibitor of miR-29b was a 2′-*O*-methyl-modified oligoribonucleotide single strand with the sequence 5′-AACACUGAUUUCAAAU GGUGCUA-3′. miRNAs (20 nM) were transient transfected for 48 hrs using G-fectin solution (Genolution Pharmaceuticals, Inc., Seoul, South Korea) according to the manufacturer's instruction.

### Ionizing irradiation treatment of cells

Cells were plated in 100 mm dishes and incubated at 37°C under humidified 5% CO_2_-95% air in culture medium until cells reached 40–50% confluent. Ionizing radiation (IR) was performed to 50% confluent cells as 10 Gy and do further experiment at 1 day after IR. Cells were then exposed to γ-rays with 137Cs γ-rays source (Atomic Energy of Canada, Ltd, Canada) with a dose rate of 3.81 Gy/min.

### Western blot analysis

Harvested cells were subjected in RIPA buffer and add to protease inhibitor cocktail tablet (Roche, Indianapolis, IN, USA). Total protein extract was separated by sodium dodecyl sulfate (SDS)-polyacrylamide gel electrophoresis (PAGE), electro-transferred to the PVDF membrane (Millipore Corporation, Billerica, MA, USA) and blocked in 5% skim milk in TBST (10 mM Tris-HCl, pH 8.0, 150 mM NaCl and 0.05% Tween 20). The indicated primary antibody were reacted as 1:1000~5000 for overnight at 4°C. The secondary antibody, mouse, rabbit and goat were reacted as 1:5000~10, 000 for 2 hrs at room temperature and detected chemiluminescence with an enhanced chemiluminiscence system (WesternBright™ ECL, Advansta, Menio Park, CA).

### Quantitative real-time reverse transcriptase-PCR

Total RNA from culture cells was isolated using RNA extraction kit (Favorgen Biotech., Ping-Tung, Taiwan) according to the manufacturer's instruction. The first strand cDNA was synthesized by reverse transcription and was amplified using RCR cycler (DNA Engine Opticon^®^2 system, Bio-Rad laboratories, Inc., Herculues, CA, USA). The expression level of miR-29b was quantified by real-time qRT-PCR using Mir-X miRNA qRT-PCR SYBR kit (Clontech Laboratories Inc., Mountain view, CA, USA). Primer of miR-29b was purchased from as follows; 5′-UAG CAC CAU UUG AAA UCA GUG UU-3′. The cycle threshold (Ct) values are similar to within 0.5 among triplicates. The primer was designed for miR-29b and U6, which yielded a 2^−ΔΔCt^ value and used for normalization. The experiments were performed in triplicate.

### Transwell invasion assay

These assays were performed as described previously [8]. Tumor cell invasion was measured by examined cells (2 × 10^5^) in 100 μl of medium were seeded onto the upper surfaces of Matrigel-coated polycarbonate filters which were coating with ECM components (BD Bioscience, Bedford, MA). These inserts were then placed in modified Boyden chambers (Corning, Corning, NY) and the lower compartment was filled with 1 ml of serum-free media containing with 0.1% bovine serum albumin. After 18 hrs of incubation at 37°C incubator, the migrated cells move to the lower surface of the filter, these cells were fixed, stained using Diff-Quick Kit (Fisher Scientific, Pittsburgh, PA), dried well and counted under a light microscope (Mitoti AE31 series, Trinocular inverted MIC) [10].

### Wound healing assay

Wound healing assays were undergone using same procedure but with normal cell culture dish. The confluent monolayer cell was scratched using yellow tip, washed with PBS 3 times to remove cell debris (or mass) and then incubated for 16–24 hrs at 37°C incubator. The cells were washed with PBS twice, fixed with methanol: acetic acid (3:1) for 30 mins followed by staining with 0.05% crystal violet in 10% ethanol for 30 mins at RT with rocking and washed with tap water. Cell in five fields in the scratched area (200 × 500 μm^2^) were counted under a light microscope (Mitoti AE31 series, Trinocular inverted MIC) [36].

### Reporter assay

U251 cell was seeded into 24-well culture plates, reached to around 50% confluency and then co-transfected for 48 hrs with reporter plasmid (200 ng), pRL-CMV-*Renilla* (Promega, Madison, WI) plasmid (1 ng) and miRNA using Lipopectamine 2000 (Invitrogen). Luciferase activity was measured using dual-luciferase reporter Assay system (Promega) according to the manufacturer's instructions [22] and normalized to *Renilla* luciferase activity. All experiments were performed in triplicates.

### Neurospheres culture and neurosphere formation assays

U251 and U87 cells was suspended in Dulbecco's modified Eagle's medium-F12 (Cellgro, Manassas, VA) containing B27 (1:50) (GIBCO, USA) as cancer stem cell permissive medium [36]. After miR-C, miR-29b, anti-miR-control, and anti-miR-29b transfected into U251 and U87 cells for 24 hrs, transfectant cells were detached with trypsin-EDTA, counted cell numbers (1 × 10^3^), and then resuspended onto 100 mm cell dish for 5–10 days. The medium was added 1 ml every 3 day. The colony was grown and tested for the sphere formation assay. After 7 days, sphere were attached by 15% added FBS for 1 days and stained with Coomassie Brilliant Blue R-250 solution (BioWorld, USA). Spheres were counted with a diameter > 20 μm under an inverted microscope (Miotic AE31 series) [11].

### Tube formation assay

This method was underdone as described previously [19]. miR-C, miR-29b, anti-miR-C, and anti-miR-29b were transfected into HUVECs for 24 hrs, incubated them without serum free medium for 24 hrs, and transfectant cells were detached by trypsinization. The 96-well plate was coated with 50 μl growth factor-reduced Matrigel (BD Bioscience). HUVECs (4 × 10^4^) were resuspended in 100 μl of conditioned media with 1% FBS and seeded on Matrigel-coated 96-well plate. HUVECs were incubated for 8 hrs to allow formation of tube-like structures. Number of formed tube were counted and compared from three different fields under a light microscope. Three independent experiment, were analyzed for statistical significance using the student's *t*-test. Differences were considered to be statistically significant at *, *p* < 0.05.

### Gelatin zymography

To measure the secreted MMPs, this method was used. Conditioned media were prepared by incubating cells in serum-free media for 24 hrs after transient transfection for 24 hrs, detached cells and attached cell into 24-well plates. After collection of conditioned media, equal volume of these media was loaded to 8% SDS-PAGE containing 0.1% gelatin as 75 voltages. The gels were stained and the MMPs activities were visualized as clear band on the white box [8]. Ponceau S staining is as the loading control.

### Statistical analysis

All results were analyzed for statistical significance that was assessed using a Student's *t*-test. A *P* value of *p* < 0.05 compare with the control was considered statistically significant.
